# Simulation study of a low‐tech and reliable identification coding method for mass casualties

**DOI:** 10.1002/ams2.342

**Published:** 2018-04-26

**Authors:** Soichiro Kato, Takehiko Tarui, Yoshihiro Yamaguchi

**Affiliations:** ^1^ Department of Trauma and Critical Care Medicine Kyorin University School of Medicine Mitaka‐city Tokyo Japan

**Keywords:** Emergency responders, information management, mass casualty incidents, triage

## Abstract

**Aim:**

To evaluate the ease of use, reliability, and duplication risks of a new identification (ID) coding method, which works for mass casualty events such as disaster scenes.

**Methods:**

The new ID code consisted of 16 alphanumeric characters: seven characters for the responder's information and nine for the casualty, which can be created in a very low‐tech manner without using any electronic devices. A simulated triage was carried out for virtual causalities by students of the same grade at our university's medical school. Each participant was instructed to triage and create IDs for the same 10 virtual casualties. Eighty‐nine participants created a total of 890 IDs, which were examined for correct coding and ID duplication rates.

**Results:**

Despite situations in which the risk of duplication might be considered high, complete duplication of IDs occurred in only one case (0.2%), and the other 888 IDs (99.8%) were unique. The simulation was done in a reasonable amount of time without any confusion.

**Conclusions:**

In the mass casualty incident triage simulation, our new coding method proved easy and useful in creating IDs with an extremely low duplication rate. To develop this method for broader use, further evaluation is needed in more simulations and real disaster situations.

## Introduction

Over the last two decades, there have been frequent mass casualty incidents (MCIs) worldwide, such as the September 11 attacks in which approximately 3,000 people died in the USA^1^ and the Great East Japan Earthquake in which approximately 19,000 people died or were lost in Japan.^2^ Therefore, people around the world are worried about future catastrophic events, such as earthquake and terrorist attacks at mass gatherings. The development of a reliable information‐sharing system covering the large number of casualties from a disaster or MCI is needed.

Management of a large‐scale disaster requires numerous organizations, including hospitals, fire departments, government agencies, police, and military (the self‐defense force in Japan) to coordinate. Given the confusion in the acute phase of a disaster, accurate sharing of casualties’ information is crucial in disaster management. To achieve effective information sharing at disaster sites, it is essential to use a standard method of recording accurate personal identification without the risk of duplication. However, it could be difficult at disaster sites to obtain personal information from semi‐conscious casualties, small children, foreigners, and the elderly, as they may lack the ability to provide accurate information including name, age, address, and medical history. Such details are important not only for casualties and evacuees in disasters but also in emergency medical and other risk‐management situations.^1^, ^3^, ^4^ This issue has a profound effect on communication among accident scenes, hospitals, and other organizations.^2^, ^5^, ^6^ From this point of view, a personal identification (ID) code that is allocated in a uniform way by first responders is one of the solutions.

Currently, many recording techniques during disasters exist around the world. Triage tags are one such technique,^7^, ^8^, ^9^, ^10^ but according to an Australian study comparing triage tag techniques, there is no recommendation on which type of triage tag should be used.^7^, ^8^ Most recording techniques do use a numbering system. However, there is no worldwide standard rule on the coding method used at disaster sites. The majority use either prearranged numbers with barcodes or simply number in order of registration, making it sometimes difficult to share information with other systems that are using different numbering systems, while also involving a risk of duplicating IDs among the different systems. For instance, a joint working group of Japanese emergency response agencies proposed a medical record format using a 16‐character ID code at disaster scenes.^11^ This coding method requires information on date of birth and full name written in Japanese unique characters. However, the way in which an unknown name or date of birth is dealt with is complicated, and by using Japanese characters, the created ID is difficult to use in other systems. Another potential method is to use the coordinates of the location where casualties are discovered, which is not practical in disaster situations as a specific tool is required. Methods using special tools or techniques incompatible with other recoding media lack certainty in situations of great devastation. Furthermore, high‐tech methods to create and decode the IDs cannot be utilized either in those disastrous occasions. Therefore, if there were more certain and uniform codes allocated by first responders who triage the casualties at the scene using a low‐tech method, we consider this could be a solution. Once such an ID is created at first contact, accurate information sharing should then be possible. In this study, we developed and verified a simple ID coding method, which can be created in a low‐tech manner, and evaluated its ease of use, reliability, and risk of duplication.

## Methods

We developed and verified a low‐tech and reliable ID coding method for casualties, allocated by emergency responders at first contact. We evaluated the coding method in terms of its ease of use and risk of different responders creating duplicate IDs.

### Identification coding method

**Figure 1:**
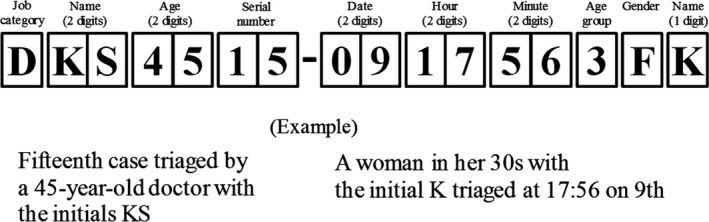
An example of a 16‐character code for identification and triage during mass casualty events such as disaster scenes. The left‐ and right‐hand sides of the code, separated by the hyphen, provide information on the responder and the casualty, respectively.

On first contact with each casualty, the responder creates an ID consisting of 16 alphanumeric characters and enters it on the triage tag (Fig. 1).

The ID consists of seven alphanumeric characters on the left for information on the responder and nine alphanumeric characters on the right for information on the casualty. The responder's information includes job category (one character), initials (two characters), age (two characters), and serial triage number in the disaster (two characters). The casualty's information includes the time of triage (date, hour, and minutes; two characters each), age group (one character), gender (M or F; one character), and initial (one character). Any information that could not be obtained from casualties is represented by “u” (“unknown”) in the space to reduce coding time.

Actual job categories of those performing triage at disaster sites may be slightly different across countries. In this coding method, we used four job categories used by the triage tags of the Fire and Disaster Management Agency in Japan: doctor (D), nurse (N), paramedic (P), and volunteer (V). The serial triage number is written as follows: first case is 01, 100th case is 00, and 101st case is 01 again. The time of triage is estimated on occasions where the responder has no watch. Ten digits are used for the casualty's age groups: if the casualty is <10 years old, his or her age group is written as 0. In the same manner, 65 years old is written as 6, and 90 years old or above as 9. When casualty's accurate age cannot be obtained, estimated age is used for this column. Gender is written as M for male and F for female.

### Participants

The participants comprised 91 voluntary students from the fourth grade of our university's medical school, none of whom had prior experience in triage, including training, or in disaster medical care. The notification that the simulation never involved any grading of participants as students, was presented before they participated. All participants agreed to analyze their initials, age, and created IDs in submitted documentation. In addition, we planned to analyze only initials, age, and created IDs from participants and to handle their personal information carefully to protect their privacy. The participants were not identified as individuals from results of this analysis. Therefore, we did not submit this study to the ethical committee.

### Verification methods

**Figure 2:**
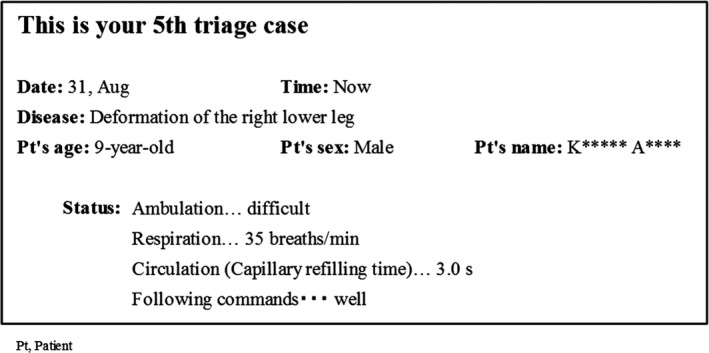
An example of a virtual casualty's information sheet used to test the ease of use, reliability, and duplication risks of a new identification coding method for mass casualty events.

Before performing the task, the participants were lectured for approximately 15 min on the significance of triage and its application at disaster sites. The task was then presented as written materials. As all the participants were students, they were assigned fictitious jobs related to disaster management, but their actual names by initials and ages were used for the coding. To make situations more liable to duplicate IDs, the serial number, date, and personal information of casualties were predetermined. After providing handouts detailing the information about casualties to each participant, the desk‐based simulation was carried out by completing the task from the top of the list all together. Table 1 shows the list of cases and Figure 2 shows an example of information about a casualty.

**Table 1:** Characteristics of virtual casualties used to test the ease of use, reliability, and duplication risks of a new identification coding method for mass casualty events

Casualty	Date	Time	Triage number	Age, years	Gender	Name	Primary injury	Classification of triage
1	31 Aug	Now	1	55	M	K***** T***	Thoracic trauma	I
2	31 Aug	Now	5	9	M	K***** A****	Deformation of the right lower leg	I
3	31 Aug	Now	11	70s	F	Unknown^a^	Head injury	I
4	31 Aug	Now	35	80s	M	T*****^b^	No injury (dementia)	III
5	31 Aug	Now	50	30s	Unknown	Unknown^a^	Extensive burns (no vital signs)	O
6	1 Sep	Now	75	55	M	N******* K******	Abdominal trauma	II
7	1 Sep	Now	99	17	M	M******** M***	Trapped right lower leg	I
8	1 Sep	Now	100	28	F	K***** S****^c^	Head injury	III
9	1 Sep	Now	101	53	F	K*** S*****	Burns to the respiratory tract	II
10	1 Sep	Now	123	28	F	K***** S****^c^	Heatstroke	I

a No name information available.

b With information of family name only.

c Persons with fully duplicated name, age and sex.

F, Female; M, Male.

O, Black; I, Red; II, Yellow; III, Green (Classified by START method).

**Figure 3:**
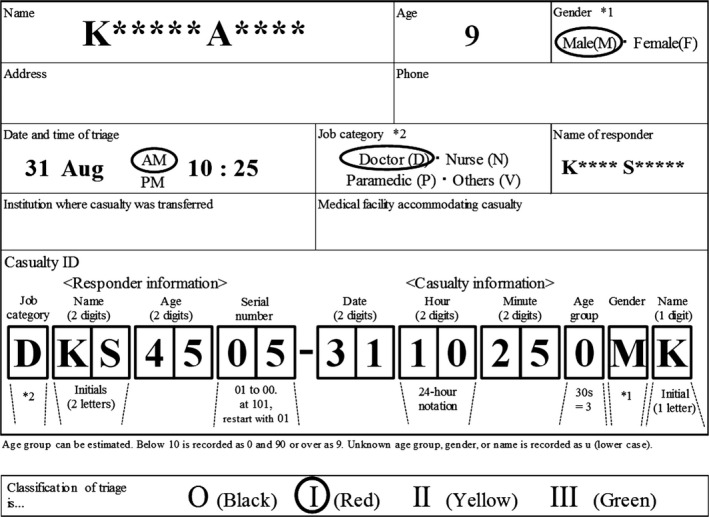
Triage format used in this study.

The participants performed the triage task using the Simple Triage and Rapid Treatment method and ID coding, simultaneously, and the real time was recorded as that of the triage. For this study, the triage tag form of Japan's Fire and Disaster Management Agency was used with a few revisions, including the addition of the new ID code (Fig. 3).

### Contents of verification

Information on the virtual casualties is listed in Table 1. Each participant triaged the same 10 virtual casualties, and was instructed to do so in the same order. Casualties with the same name or age, or whose name, gender, or age were incomplete, were included in the list, as such problems are common in MCIs. Valid responses were obtained from 89 out of 91 participants; two were excluded from the analysis because they failed to follow the instructions. The exclusion was due to their simple technical errors, which were not relevant to the reliability of this coding method. The median age of the 89 participants was 23 years (interquartile range, 22–24 years), which was considered to be a narrower range than that of actual emergency responders working at disaster sites. We therefore examined a total of 890 IDs and calculated the correct ID coding and duplication rates.

## Results

The results of the verification are shown in Table 2. All 89 participants completed the triage task, including ID coding, within 20 min at most. Examination of the created IDs revealed that the duplicate pattern of two‐character name initials were 16 out of 62 combinations; 43 participants, nearly 50%, were included in those duplications. However, when combined with age, the duplication rate was reduced to approximately 19%, and to 2.2% when combined with the age and job category (Table 2). Only one complete duplication was observed in all cases (0.2%): between two participants who were both fictitious paramedics. In spite of pre‐estimated higher probability, no other duplication was observed in the remaining 18 IDs coded by these two participants. In these 18 IDs, duplications were prevented by casualty information: minutes of triage (in 14 cases) and patients’ initials (in four cases). Including these 18 IDs, 888 IDs (99.8%) were valid and effective for identification.

**Table 2:** Verification results of the desk‐based simulation of a new identification coding method for mass casualty events

Items	Median	IQR
Number of participants	89	―
Age of participants, years	23	22–24
Job category (assigned)	D 29, N 15, P 19, V 26	
Duplication of participants’ information	Number	%
Name: e.g., KS^a^ (combination pattern of initials)	43/89 (16/62)	48.3
Name and age: e.g., KS35^a^ (combination pattern of initials and age)	17/89 (6/82)	19.1
Name, age, and job category: e.g., DKS35^a^ (combination pattern of initials, age, and job category)	2/89 (1/88)	2.2
Duplication of fully created IDs	Number	%
Complete duplication of 16 characters: e.g., DKS3501‐0101010MK^a^	2/890	0.2

a Not the real duplicated characters or IDs in this study.

D, doctor; IQR, interquartile range; N, nurse; P, paramedic; V, volunteer.

The fully correct coding rate was 90.4%; the 9.6% of entries with errors were due to simple mistakes such as entering the incorrect gender and date. None of these errors were related to this coding methodology or led to or prevented the duplication of IDs.

## Discussion

This study examined the usefulness of our low‐tech ID coding method for first responders to register mass casualties at disaster sites in a desk‐based simulation. The simulation undertaken by students revealed that the new method was easy to use and had a very low duplication rate.

The coding method used in this study has the following advantages: captures both responders’ and casualties’ information; no specific tools required; and quick completion even when information is incomplete. First, our coding method consists of a combination of information about the responder and the casualty to create a unique ID without using specific tools, while containing the variable information of both the first responder and the casualty. In this study, we confirmed that participants were able to easily create IDs with an extremely low rate of duplication. There are a number of last names that are shared by a large proportion of the population in Japan, for example, Sato, Suzuki, and Saito. In addition, there are some popular first names that are shared by a significant number of people in each generation, for example, Tomoko and Takako are common female names, and Tatsuya and Takuya are common male names. When we recruit participants with these names, all of their initials are going to be “TS”. The same situation may occur in other countries, for example, Zhang and Zhao are popular last names in China, and Johnson and Jones as common in the USA. Although the initials of participants were frequently duplicated in our simulation, our method effectively reduced the duplication of responders’ information (Table 2). Furthermore, we consider this method can be introduced without changing the major parts of existing recording formats. It only uses numbers and Roman characters, and is therefore available for international use and electronic media. This coding method also assures quick completion even when the information required is not available. For example, in this coding, the method of entering “u” in the space of unknown characters for casualties’ information saves time for quick coding. Moreover, the first five characters of this ID, indicating personal information of the responder, can be filled in with certainty and completed beforehand to save time at disaster sites. If computerized, automated recordings of this predetermined information and the actual time of triage can lead to further simplification and speeding up of the procedure. Therefore, the proposed ID can be a robust tool for accurate information dissemination and communication among the various entities involved in disaster management.

Instead of being seen as an advantage, there are concerns that there may frequently be same initials in some countries or regions in which there are many persons with the same family name or same personal name. To verify the usefulness of this coding method from this point of view, we set the condition that participants were prone to duplicating IDs: participants were recruited from the same grade (narrow age range), there were 10 virtual casualties that included with the same name and age, or unknown details, and all participants performed the triage task simultaneously in the same order. As a result, the complete duplication rate of IDs was found to be low at 0.2%. Duplicate IDs could be mostly avoided by incorporating job category and age of responder. When combined with information on the casualties, the ID duplication rate was further diminished, and was almost completely avoided when further combined with the triage number and date. Although the risk of duplication was not zero, we expect that the risk would be extremely low in real practice. It is because there are fewer responders at small‐scale disasters, whereas the time frame is longer in large‐scale disasters. In addition, some part of the simulation in this study became intentionally confusing due to designed intention to produce duplicate entries for verification. For that reason, a 9.6% rate of coding error occurred. However, as stated in the results section, the errors were not related to this coding methodology and did not lead to the duplication of IDs; in fact, all of the IDs with errors had still worked for identification. Thus, we consider that this coding method is reliable even in disaster scenes in which IDs are prone to duplication.

Our study has some limitations. This was a simulation study carried out by students with no experience as emergency responders or in disaster management. As the types of casualties were limited, the task was much easier than in real disasters: real responders have to deal with a wide range of disaster‐related presentations, including triage and first aid, in unusual circumstances. It is also necessary to make sure that this can be used in the field environment, such as in bad weather. Therefore, more practical training that simulates large‐scale disasters or MCIs is required before implementing this method into practice during real disasters. For this future use, we are planning to develop a “casualty ID label” to attach to existing triage tags (see the draft version in Fig. 1). After a number of verifications, we hope that current ID codes on triage tools, including tags, would be replaced with those using this method in the near future.

## Conclusions

We developed and verified a low‐tech and reliable ID coding method for casualties at disaster sites. The simulated MCI revealed that the new method was easy to use and had a low duplication rate. Further verification of the method in simulations and in real disasters is warranted.

## Disclosure

Approval of the research protocol: N/A.

Informed consent: Informed consent was obtained from all participants.

Registry and registration no. of the study/trial: N/A.

Animal studies: N/A.

Conflict of interest: None declared.
